# Uric Acid Potential Role in Systemic Inflammation and Negative Symptoms After Acute Antipsychotic Treatment in Schizophrenia

**DOI:** 10.3389/fpsyt.2021.822579

**Published:** 2022-02-14

**Authors:** Milica M. Borovcanin, Slavica Minic Janicijevic, Natasa R. Mijailovic, Ivan P. Jovanovic, Nebojsa N. Arsenijevic, Katarina Vesic

**Affiliations:** ^1^Department of Psychiatry, Faculty of Medical Sciences, University of Kragujevac, Kragujevac, Serbia; ^2^Doctor of Philosophy Studies, Faculty of Medical Sciences, University of Kragujevac, Kragujevac, Serbia; ^3^Department of Pharmacy, Faculty of Medical Sciences, University of Kragujevac, Kragujevac, Serbia; ^4^Center for Molecular Medicine and Stem Cell Research, Faculty of Medical Sciences, University of Kragujevac, Kragujevac, Serbia; ^5^Department of Neurology, Faculty of Medical Sciences, University of Kragujevac, Kragujevac, Serbia

**Keywords:** uric acid, schizophrenia, cytokines, interleukin-6, interleukin-17

## Abstract

Uric acid (UA) has been shown to have neuroprotective or neurotoxic properties, in relation to specific tissues and diseases that have been studied. Previous studies provided contradictory results on the role of UA in schizophrenia as a neurodegenerative disorder. The aim of this brief report was an additional analysis of UA sera levels in different phases of schizophrenia. Here, 86 patients with first-episode psychosis (FEP) vs. 45 patients with schizophrenia in relapse (SC in relapse) vs. 35 healthy control subjects (HC) were studied before and 1 month after antipsychotic therapy. Further, we aimed to explore the possible correlation of UA with scores presenting clinical features and with serum concentrations of the proinflammatory cytokines interleukin (IL)-6 and IL-17. When comparing the data between all three groups, we did not find significant differences in UA levels, either before or after the applied therapy. Also, comparing sera concentrations of UA in every single group, the analysis did not reveal statistically significant differences between FEP patients, but statistically, a significant difference was found in SC in relapse before and after treatment (334.71 ± 116.84 vs. 289.37 ± 109.15 μmol/L, *p* = 0.05). Uric acid serum levels correlated with negative sub-score (*p* = 0.001, *r* = 0.306), general sub-score (*p* = 0.015, *r* = 0.236), and total PANSS score (*p* = 0.009, *r* = 0.3) after 1 month of therapy. We have established a statistically significant positive correlation between serum concentrations of UA and IL-6 in exacerbation (*p* = 0.01, *r* = 0.220) and with IL-17 after treatment and in the stabilization of psychosis (*p* = 0.01, *r* = 0.34), suggesting potential cascades in different phases of schizophrenia that potentiate inflammation.

## Introduction

Uric acid (UA) is the final oxidation product of the adenine- and guanine-based purine catabolic pathway and is considered as a risk factor of numerous pathological conditions (1). Homeostatic imbalance of purine catabolism has been associated with various psychiatric disorders, such as depression (2, 3), schizophrenia (4), and bipolar disorder (5). It has been postulated that UA may exert both neuroprotective and/or neurotoxic effects in the brain tissue (6), by modulating oxidation processes and inflammatory response, which could be of particular interest in the pathophysiology of schizophrenia. In a recent review paper, Morris and Maes (7) detailed that the immune-inflammatory response is associated with increased nitro-oxidative stress through the activation and involvement of macrophages, dendritic cells, neutrophils, T cells, B cells, and natural killer cells, is associated with increased nitro-oxidative stress. These redox-associated mechanisms modulated the production of pro- vs. anti-inflammatory cytokines.

Increased levels of UA in the initial phase may reflect an attempt to counteract oxidative stress, suggesting its neuroprotective role (8), whereas the sustained increase in UA may also be considered a marker of oxidative stress (4), and could lead to a systemic inflammatory response (4). Uric acid is thought to have strong proinflammatory activity by triggering interleukin (IL)-1β-mediated inflammation via activation of the nucleotide oligomerization domain-like receptor protein (NLRP) 3 inflammasome, a multimolecular complex that plays a central role in many pathological inflammatory conditions (9, 10). In neuroprogressive disorders, UA may act as an alarmin, leading to NLRP3 inflammasome activation, and internalized UA may increase nuclear factor-κB activity as well as oxidative, nitrosative, and inflammatory stress (7). Elevated UA levels correlate positively with several pro-inflammatory markers such as C-reactive protein and white blood cells count (11), IL-6, IL-17, tumor necrosis factor-alpha (TNF-α) (11). The role of IL-6 has also been discussed in the etiopathogenesis of schizophrenia in terms of treatment-resistant schizophrenia being associated with increased IL-6 serum levels, an association between higher IL-6 levels and cognitive decline, that was established, and a decrease in serum levels of IL-6 following antipsychotic therapy (12). Recently, we have observed that the predomination of type 17 immune response facilitates the progression of inflammation and could be involved in the regulation of cognition (13).

The relationship between altered UA levels and schizophrenia remains unclear, and studies have yielded conflicting results. A significant decrease in plasma UA levels was observed in the first episode of schizophrenia (14), and a meta-analysis confirmed reduced UA levels in patients with first-episode psychosis (FEP) (15). Previous studies also revealed that schizophrenia patients had lower UA levels compared to healthy control (HC) subjects (4, 16), whereas several studies reported higher UA levels in schizophrenia patients compared with HCs (17, 18). However, it is still not known whether the reduction in UA levels occurs early in the course of the illness and whether it is independent of treatment or illness progression. This study aimed to examine whether serum levels of UA are altered in drug naïve patients with FEP and in patients with schizophrenia in relapse (SC in relapse) and compared them with levels measured in HC subjects. A secondary aim was to assess the effects of acute treatment on UA serum levels after 1 month. Finally, we aimed to explore the correlations between UA and the cytokines IL-6 and IL-17 and to further test whether altered UA serum levels are associated with specific clinical features of schizophrenia.

## Materials and Methods

### Subjects

This study represents an additional analysis of the database from our previous cytokine studies in different phases of schizophrenia (19, 20). Briefly, patients were enrolled in Psychiatric Clinic, Clinical Center Kragujevac, and the Special Hospital for Psychiatric Diseases “Dr. Laza Lazarevic,” Belgrade. We examined 86 drug-naive patients with FEP, 45 patients with SC in relapse who were already treated with antipsychotics, and 35 HC subjects. The International Statistical Classification of Diseases and Related Health Problems, Tenth Revision (ICD-10) (21) was used to determine the diagnoses. The psychopathological status of psychotic patients was assessed by trained physicians using the Positive and Negative Syndrome Scale (PANSS) (22). Exclusion criteria included acute or chronic medical conditions, acute infections, recent surgical procedures, alcohol or drug abuse, any illness that is immune-and redox-mediated, allergies or autoimmune disorders, ongoing anti-inflammatory or antiviral treatment, and treatment with medications that can influence UA levels (vitamin C, diuretics, epinephrine, levodopa, methyldopa, acetylsalicylic acid, and azathioprine). The research project was approved by two ethics committees of these institutions and was conducted in compliance with the ethical principles of the Declaration of Helsinki. Written informed consent was obtained from all patients prior to any study procedure.

### Laboratory Measurements

A somatic examination was performed, and vital signs were measured on admission. Patients' blood samples were collected at least 8 h before the administration of antipsychotic therapy during the current psychotic episode. The control group consisted of HC subjects matched to the patients by gender and age with patients and recruited at a blood donation in the Service supply of blood and blood products, of Clinical Center Kragujevac. Serum UA levels were determined using a hematology analyzer, ABX MICROS 60-OT (UK). The reference values for UA were in the following range: 154–428 μmol/L. Serum cytokine levels were measured using conventional enzyme-linked immunosorbent assay (ELISA) kits (R & D Systems Minneapolis, MN for IL-17 and IL-6, according to the manufacturer's instructions). Immunological measurements were performed at the Center for Molecular Medicine and Stem Cell Research, Faculty of Medical Sciences, University of Kragujevac. The acute effects of treatment on UA serum levels were assessed after 4 weeks, since the first 2–4 weeks of antipsychotic treatment are crucial for a substantial reduction of positive symptoms (23).

### Statistical Analysis

Statistical analyses were performed using SPSS 20.0 software (SPSS Inc., Chicago, Illinois, USA). Numerical values are reported as mean, standard deviation (SD), and standard error (SE). The normal distribution of the data was tested using Shapiro–Wilk and the Kolmogorov–Smirnov test. A Paired Sample test and the Wilcoxon test were used to compare the clinical parameters in the same group before and after therapy. We used the Mann–Whitney test for non-parametric variables to assess mean differences between two groups of patients. A Kruskal–Wallis test was used to assess differences in serum UA levels between three groups (FEP, SC in relapse, and HC). We examined the correlations between serum UA and cytokines, as well as the relationship between serum UA levels and PANSS scores, positive, negative, and general psychopathology sub-scores using Pearson's or Spearman's correlation. Multiple regression analysis was used to assess the significant demographic and clinical predictors of serum UA levels and PANSS scores. A *p-*value of 0.05 was considered to be statistically significant.

## Results

From our previous study database (19, 20), only patients with successful UA sampling were included. Sample characteristics were as expected in terms of phase of the disease and applied therapy (see Table 1 for details).

**Table 1:** Demographic and clinical characteristics of the sample.

**Parameters**	**FEP**	**SC in relapse**	**Controls**	
	***n*** **= 86**	***n*** **= 45**	***n*** **= 35**	
**Age (years, mean ± SD)**	33.64 ± 8.84	35.95 ± 11.40	36.63 ± 8.73	
**Sex (male/female)**	36/50	17/28	22/13	
**Mean duration of illness (mean ± SD)**	0.28 ± 1.93	7.31 ± 6.30	NA	
**Positive and Negative Syndrome Scale (PANSS) (mean ± SD):**				
	**Day 0**	**Day 30**	**Day 0**	**Day 30**
Total score	100.96 ± 14.76	56.22 ± 21.01^**^	104.39 ± 18.88	65.82 ± 21.45^**,***^
Positive symptoms	25.49 ± 5.74	12.36 ± 5.37^**^	25.97 ± 6.50	18.35 ± 24.3^***^
Negative symptoms	22.08 ± 5.78	13.15 ± 5.86^**^	24.09 ± 8.27^*^	16.82 ± 7.59^**,***^
General psychopathology	53.40 ± 7.13	30.71 ± 11.00^**^	54.55 ± 8.24	34.76 ± 10.82^**,***^

* *Kruskal–Wallis test, statistically significant difference between two groups at the baseline (p = 0.006)*.

** *Paired sample test, statistically significant difference between two measurements (p < 0.005)*.

*** *Kruskal–Wallis test, statistically significant difference between two groups after antipsychotic therapy (p = 0.05)*.

When comparing data between all three groups of FEP, SC in relapse and HC subjects (339.04 ± 175.97 vs. 334.71 ± 116.84 vs. 332.60 ± 83.71 μmol/L, *p* = 0.663) and after applied therapy (304.01 ± 113.06 vs. 289.37 ± 109.15 vs. 332.60 ± 83.71 μmol/L, *p* = 0.349), we did not detect statistically significant differences in UA values before therapy. When comparing serum UA levels in each group, the analysis did not reveal statistically significant differences between FEP patients (339.04 ± 175.97 vs. 304.01 ± 113.06 μmol/L, *p* = 0.207), but statistically, a significant difference was found in SC in relapse, before and after treatment (334.71 ± 116.84 vs. 289.37 ± 109.15 μmol/L, *p* = 0.05). We also analyzed serum UA levels between FEP and SC in the relapse group before treatment (339.04 ± 175.97 vs. 334.71 ± 116.84 μmol/L, *p* = 0.611), and after treatment (304.01 ± 113.06 vs. 289.37 ± 109.15 μmol/L, *p* = 0.349), and no statistically significant differences were observed (presented in Table 2).

**Table 2:** Comparison of serum uric acid levels between groups.

**Parameter**	**FEP**	**SC in relapse**	**HC**	***p***
**Uric acid**			
Before treatment (mean ± SD)	339.04 ± 175.97	334.71 ± 116.84	332.60 ± 83.71	0.663
After treatment (mean ± SD)	304.01 ± 113.06	289.37 ± 109.15	332.60 ± 83.71	0.349
**Uric acid**			
	**Before treatment (mean ± SD)**	**After treatment (mean ± SD)**		***p***
FEP	339.04 ± 175.97	304.01 ± 113.06		0.207
SC in relapse	334.71 ± 116.84	289.37 ± 109.15		0.050^*^

*FEP, first-episode psychosis; SC in relapse, schizophrenia in relapse; HC, healthy controls*.

* *Wilcoxon test, statistical significance p < 0.05*.

**Figure 1:**
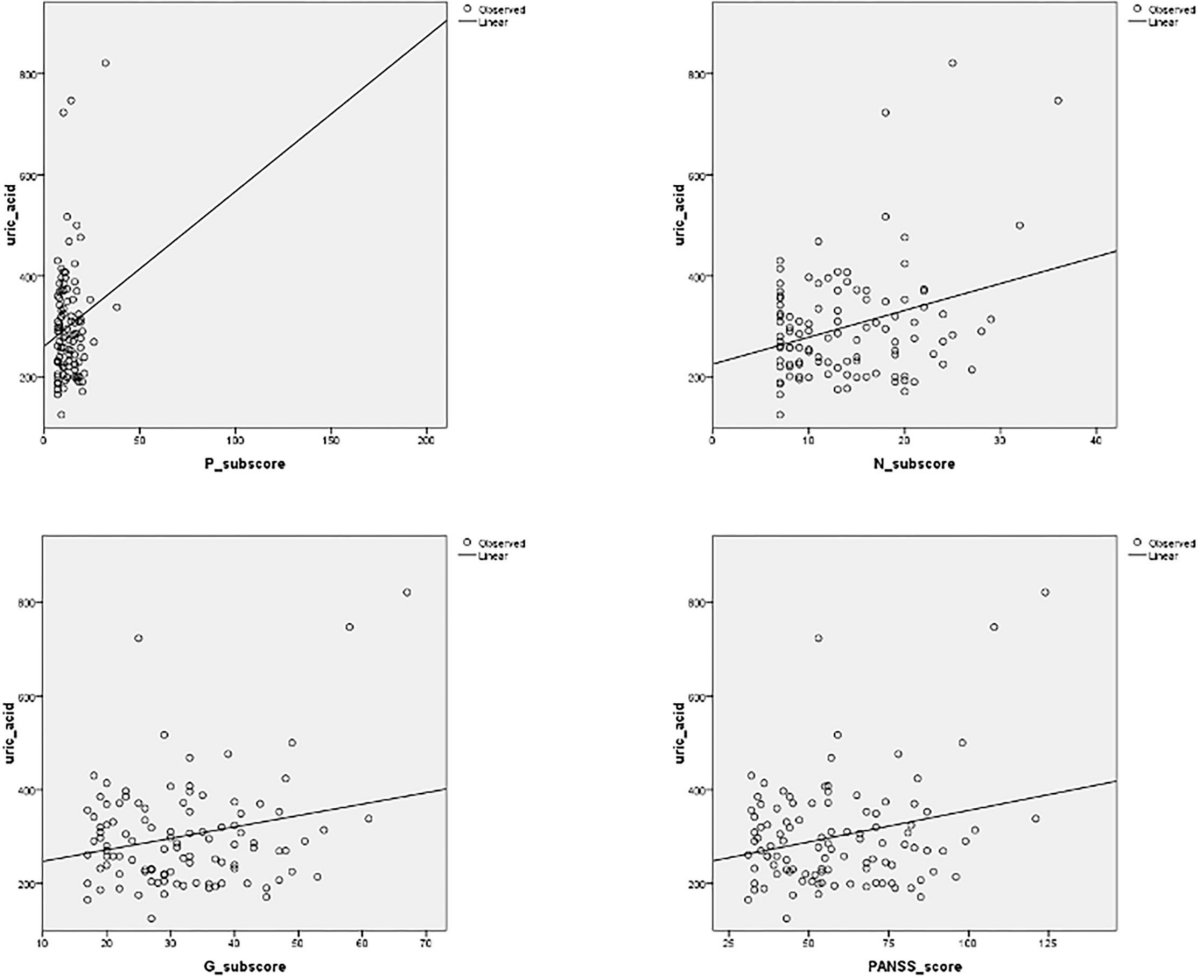
Correlation between serum uric acid (UA) levels and positive, negative (N), and general (G) sub-scores, and total PANSS (Positive and Negative Syndrome Scale) score, after 1 month of antipsychotic treatment. Significant correlation was established between UA serum levels with negative sub-score (*p* = 0.001, *r* = 0.306), general sub-score (*p* = 0.015, *r* = 0.236), and total PANSS score (*p* = 0.009, *r* = 0.3) after 1 month of therapy.

We found a significant correlation between UA serum levels with negative sub-score (*p* = 0.001, *r* = 0.306), general sub-score (*p* = 0.015, *r* = 0.236), and total PANSS score (*p* = 0.009, *r* = 0.3) after 1 month of therapy in both patient groups, while a correlation between serum UA levels and positive sub-score was not significant (*p* = 0.128, *r* = 0.149) (Figure 1). In addition, we found a statistically significant, but weak positive correlation between serum concentrations of UA and IL-6 in exacerbation (*p* = 0.01, *r* = 0.220) and with IL-17 after treatment and in the stabilization of psychosis (*p* = 0.01, *r* = 0.34) (Table 3).

**Table 3:** Positive correlation of UA with IL-6 serum levels before, and with IL-17 serum levels after acute treatment.

**Serum concentration (pg/ml)**	**Uric acid**	
	**Spearman's rho**	***p***
**IL-6**	0.220	0.01^*^
**IL-17**	0.340	0.01^*^

* *Spearman's correlation, statistical significance p < 0.05*.

Multiple regression analysis indicated that sex [*F*_(3/108)_ = 8.027, *p* < 0.001, *R*^2^ = 0.187, Adjusted *R* = 0.163] was a significant predictor of serum UA levels, with female gender implicating an increase in UA. Uric acid serum levels [*F*_(4/101)_ = 2.192, *p* = 0.013, *R*^2^ = 0.080, Adjusted *R* = 0.043] and IL-17 serum levels after treatment in all psychotic patients [*F*_(1/48)_ = 5.027, *p* = 0.030, *R*^2^ = 0.097, Adjusted *R* = 0.077] are significant predictors for PANSS score (presented in Table 4).

**Table 4:** Results of multiple regression analysis with serum UA levels and total PANSS score in all psychotic patients after therapy as dependent variables and demographic and clinical data as explanatory variables.

**Dependent variables**	**Explanatory variables**	***B***	***SE***	**β**	***T***	***P***
**UA levels**	Sex	72.671	15.657	0.410	4.641	<0.001^*^
**PANSS score**	UA	0.052	0.020	0.268	2.534	0.013^*^
	IL-17	0.155	0.069	0.311	2.242	0.030^*^

*UA, uric acid; PANSS, Positive and Negative Syndrome Scale; IL-17, Interleukin-17*.

* *Statistically significant p < 0.05*.

## Discussion

In the new tendency to publish negative results, we must emphasize that we did not find statistically significant differences in serum UA levels between patients with FEP, SC in relapse, and HC subjects, neither before nor after the applied therapy. We pointed out that patients with schizophrenia had higher UA serum levels in exacerbation than in the stable phase of this psychosis. There is a significant correlation between UA serum levels and negative, general sub-score, and the total PANSS score in the stabilization phase. A statistically significant positive correlation was observed between serum concentrations of UA and IL-6 in exacerbation, and with IL-17 after treatment and in the stabilization of psychosis. Our findings suggest that in a stable phase of the illness, serum levels of UA and IL-17 are significant predictors for PANSS scores.

Firstly, we would like to discuss phase specificities of oxidative and inflammatory stress in schizophrenia. Plasma UA levels were significantly lower in patients with FEP, but not in patients with bipolar or depressive disorder (14). In contrast to our findings, a recent meta-analysis that included 17 studies revealed that UA levels were decreased in subjects with a first psychotic episode (24). In a study by Yao et al. (25), symptom severity was measured with the Bunney-Hamburg Global Rating Scale, and a significant inverse relationship was found between plasma UA levels and psychosis scores. Previous studies showed that patients with schizophrenia had lower UA levels compared to HCs (4, 16). Other studies found no differences in UA levels in schizophrenia patients when comparing the acute and remission phases of the illness (26). In contrast, several studies reported higher levels of UA in patients when compared with HCs (17, 18). Our results are consistent with the most recent meta-analysis from 2020, which found no significant difference in UA levels between patients with schizophrenia and HCs, regardless of the use of the antipsychotics (24). To date, it is still not clear whether serum UA levels should be considered in schizophrenia onset or as an outcome of disease progression. Complex analyses of the data from genome-wide association studies within the Global Urate Genetics Consortium and the Psychiatric Genomics Consortium revealed no causal role of serum UA concentrations in schizophrenia, suggesting that low UA levels may be a consequence of schizophrenia onset and are more appropriate for diagnostic purposes or treatment monitoring (27). We examined serum UA levels in different phases of schizophrenia and found no significant difference between FEP and SC in relapse patients, nor 1 month after applied therapy in FEP. Our findings can be explained by the short duration of the disease, so we could not address possible further increases in UA serum levels in chronic schizophrenia. In patients with prolonged schizophrenia, UA levels could be initially elevated in the proinflammatory milieu during exacerbation and secondarily decrease due to the antipsychotic effect. It should be kept in mind that UA disturbances may not only be a consequence of the disease itself and aforementioned antipsychotics, but also could be a consequence of smoking (28), metabolic abnormalities such as weight gain, abdominal obesity, dyslipidemia (29, 30), hypertension (31), or insulin resistance (32).

Antipsychotics could increase the levels of UA (33). In a study designed as an on-off-on haloperidol treatment for schizophrenia, significantly lower plasma UA levels were found in male patients compared to HC subjects, and these levels decreased further after haloperidol was discontinued (25). Hyperuricemia was found to be associated with treatment with olanzapine and clozapine (34). There was no difference in plasma UA levels in patients with first-episode schizophrenia and in patients with chronic schizophrenia treated with risperidone for 6 months (35). In unmedicated patients with schizophrenia before and after 8 weeks of risperidone monotherapy, 20 marker metabolites were identified that helped distinguish between patients before and after treatment, and among them, myo-inositol, UA, and tryptophan showed the highest combined classification performance (36). In this sample, decreased serum levels of UA were normalized after risperidone treatment in responders, but not in non-responders. The uricosuric pharmacology of the antipsychotic drug and the resulting decrease in UA levels should be considered (37). Our previous publication discussed this issue of dose and type of oral antipsychotic medication in this study (20). The diversity of antipsychotic treatment hampered a more thorough analysis of the specific antipsychotic impact on UA serum levels.

In addition, the immune response seems to be modulated through UA impact. We found a statistically significant positive correlation between serum concentrations of UA and IL-6 in exacerbation and with IL-17 after treatment and in the stabilization of psychosis. Interleukin-17 is a pro-inflammatory cytokine produced mainly by Th17 cells and Type3 innate lymphoid cells when stimulated by cytokines such as IL-6 or IL-1, stimulating the production of cytokines and chemokines, and facilitating inflammation (38). An increase in Th17 cells has been reported in patients with neurodegenerative diseases (39, 40) and has also been postulated in schizophrenia. Interleukin-17 can induce blood-brain barrier disruption via a mechanism that depends on the production of reactive oxygen species, suggesting that IL-17 could exacerbate neurodegeneration through oxidative damage to lipids, proteins, and deoxyribonucleic acid (41). A recent randomized, double-blind, placebo-controlled pilot study showed a positive correlation between UA serum levels and IL-6, IL-17, and TNF-α, suggesting that xanthine oxidase inhibitors reduce levels of serum UA but also of these cytokines in patients with gout (42). It is already known that IL-6 is a key factor for Th17 differentiation and subsequent IL-17 production, and in turn, innate immunocytes respond to IL-17 by releasing IL-6. The IL-6/IL-17 axis functions as positive feedback that should be much more thoroughly explored in schizophrenia.

Yao et al. discussed the previously established links between UA and intelligence and reported the data from their research on the association between low levels of UA and greater impairment in sensory processing tasks (43). In a more recent study that included first-episode drug-free schizophrenia patients, no correlation was found between UA serum levels and scores representing cognitive functioning (44). However, we observed a weak correlation between UA serum levels and negative and general sub-scores and PANSS total score after 1 month of treatment. Coleman described the autistic child with marked hyperuricemia and also on antisocial, aggressive, or hyperactive behavior commonly observed in patients with excessive UA production (45). It has been shown that in patients with Parkinson's disease, IL-6 correlates positively with motor symptoms severity, while IL-17 correlates with non-motor symptoms, specifically mood and cognition scores, with a negative correlation reported between IL-17 and cognitive deterioration (46). Therefore, this could be similar to the changes observed in schizophrenia: excitability could be related to IL-6 and UA, and negative and cognitive symptoms might be mediated by IL-17 and UA interactions.

When examining whether these cascades might be of clinical benefit in patients with Parkinson's disease, lower UA levels seem to imply a higher risk of transition to mild cognitive impairment (47). In contrast, after adjusting for age, sex, and smoking habits, the authors found and presented significantly higher levels of UA in the stable phase of schizophrenia than in HC subjects, suggesting higher oxidative stress and greater inflammation in patients with poorer outcomes (48).

This study reflects the naturalistic sampling in real clinical practice, in a specific timeframe. The diagnosis could be made only after a longer prodromal phase of the disease, sometimes to avoid stigmatization of these patients and due to inadequate family support. The limitation of this study is also that the data do not allow extrapolation in the chronic patients and required further research of a much longer duration of the illness. In addition, the fact that the decrease in FEP patients was not statistically significant could be a by-product of the higher variance in the FEP group, which also requires a larger sample size.

Our results indicate a possible detrimental effect of elevated serum UA levels in certain phases of schizophrenia. No significant differences in UA levels were observed in the initial phase of the illness, but it has been shown by others that repeated acute relapses might be followed by increased UA levels in patients with a longer duration of illness. Uric acid serum levels could be associated with IL-6 elevation in exacerbation, once more showing IL-6 as a crossroads in the pathophysiology of schizophrenia. These results could be explained by the fact that a different type of immune response is activated in schizophrenia than in other neurodegenerative diseases. For example, multiple sclerosis predominates type 1 immune response, while in schizophrenia type 2 immune response overweights and type-17 immune response could be related to cognitive decline. It could be considered that the type of immune response influences the further role of UA and whether it exhibits pro/antioxidant or pro/anti-inflammatory effects. Also, it is important to consider the lower serum levels of UA after therapy in patients with chronic schizophrenia, and that this potential further elevation of UA may contribute to residual symptomatology and poorer therapeutical response. Uric acid can be considered as a marker of the disease course in the later disease cascades.

## Data Availability Statement

The raw data supporting the conclusions of this article will be made available by the authors, without undue reservation.

## Ethics Statement

The study involving human participants, was reviewed and approved by the Ethics Committee of the Clinical Centre Kragujevac and the Special Hospital for Psychiatric Diseases “Dr. Laza Lazarevic”, Belgrade, and was conducted in compliance with the ethical principles. Informed consent was obtained from all patients before any study procedures began.

## Author Contributions

MB and the team of trained psychiatrists have selected the patients, performed the psychological assessment, and sampling. MB has conceptualized the manuscript together with KV. KV and MB did the statistical analysis. KV designed figures and tables. MB and SJ wrote about the psychiatric aspects of this topic. KV and NM wrote about the role of UA. IJ and NA did the cytokine measurements and wrote about immunological aspects. MB prepared an integral version of the manuscript. All authors contributed equally to the content of this manuscript and approved the final version of the manuscript.

## Funding

This work was supported by the Ministry of Science and Technological Development of the Republic of Serbia, No. 175069, and by the Faculty of Medical Sciences, University of Kragujevac, Nos. JP 03/16 and JP 12/09.

## Conflict of Interest

The authors declare that the research was conducted in the absence of any commercial or financial relationships that could be construed as a potential conflict of interest.

## Publisher's Note

All claims expressed in this article are solely those of the authors and do not necessarily represent those of their affiliated organizations, or those of the publisher, the editors and the reviewers. Any product that may be evaluated in this article, or claim that may be made by its manufacturer, is not guaranteed or endorsed by the publisher.

## References

[1] El Ridi R Tallima H. Physiological functions and pathogenic potential of uric acid: a review. J Adv Res. (2017) 8:487–93. 10.1016/j.jare.2017.03.003 28748115 PMC5512149

[2] Zhou X Liu L Lan X Cohen D Zhang Y Ravindran A V. Polyunsaturated fatty acids metabolism, purine metabolism and inosine as potential independent diagnostic biomarkers for major depressive disorder in children and adolescents. Mol Psychiatry. (2019) 24:1478–88. 10.1038/s41380-018-0047-z 29679072 PMC6756100

[3] Ali-Sisto T Tolmunen T Velagapudi V Mäntyselkä P Lehto SM. Purine metabolism in patients with major depression: a follow-up study. Eur Psychiatry. (2015) 30:625. 10.1016/S0924-9338(15)31919-2

[4] Yao JK Dougherty GG Reddy RD Keshavan MS Montrose DM Matson WR. Homeostatic imbalance of purine catabolism in first-episode neuroleptic-naïve patients with schizophrenia. PLoS ONE. (2010) 5:e9508. 10.1371/journal.pone.0009508 20209081 PMC2831068

[5] Steen NE Dieset I Hope S Vedal TSJ Smeland OB Matson W. Metabolic dysfunctions in the kynurenine pathway, noradrenergic and purine metabolism in schizophrenia and bipolar disorders. Psychol Med. (2020) 50:595–606. 10.1017/S0033291719000400 30867076

[6] Jin Jun Luo XL. A double-edged sword: uric acid and neurological disorders. Brain Disord Ther. (2013) 2:109. 10.4172/2168-975X.1000109 24511458 PMC3914730

[7] Morris G Puri BK Olive L Carvalho A Berk M Walder K. Endothelial dysfunction in neuroprogressive disorders—causes and suggested treatments. BMC Med. (2020) 18:305. 10.1186/s12916-020-01749-w 33070778 PMC7570030

[8] Yu ZF Bruce-Keller AJ Goodman Y Mattson MP. Uric acid protects neurons against excitotoxic and metabolic insults in cell culture, and against focal ischemic brain injury *in vivo*. J Neurosci Res. (1998) 53:613–25. 10.1002/(SICI)1097-4547(19980901)53:5<613::AID-JNR11>3.0.CO;2-1 9726432

[9] Martinon F Pétrilli V Mayor A Tardivel A Tschopp J. Gout-associated uric acid crystals activate the NALP3 inflammasome. Nature. (2006) 440:237–41. 10.1038/nature04516 16407889

[10] Chen CJ Shi Y Hearn A Fitzgerald K Golenbock D Reed G. MyD88-dependent IL-1 receptor signaling is essential for gouty inflammation stimulated by monosodium urate crystals. J Clin Invest. (2006) 116:2262–71. 10.1172/JCI28075 16886064 PMC1523415

[11] Ruggiero C Cherubini A Ble A Bos AJG Maggio M Dixit VD. Uric acid and inflammatory markers. Eur Heart J. (2006) 27:1174–81. 10.1093/eurheartj/ehi879 16611671 PMC2668163

[12] Borovcanin MM Jovanovic I Radosavljevic G Pantic J Minic Janicijevic S Arsenijevic N. Interleukin-6 in schizophrenia—is there a therapeutic relevance? Front Psychiatry. (2017) 8:221. 10.3389/fpsyt.2017.00221 29163240 PMC5681495

[13] Borovcanin MM Minic Janicijevic S Jovanovic IP Gajovic NM Jurisevic MM Arsenijevic NN. Type 17 immune response facilitates progression of inflammation and correlates with cognition in stable schizophrenia. Diagnostics. (2020) 10:926. 10.3390/diagnostics10110926 33182582 PMC7698203

[14] Reddy R. Reduced plasma antioxidants in first-episode patients with schizophrenia. Schizophr Res. (2003) 62:205–12. 10.1016/S0920-9964(02)00407-3 12837516

[15] Flatow J Buckley P Miller BJ. Meta-analysis of oxidative stress in schizophrenia. Biol Psychiatry. (2013) 74:400–9. 10.1016/j.biopsych.2013.03.018 23683390 PMC4018767

[16] Akanji A Ohaeri J Al-Shammri S Fatania H. Associations of blood levels of insulin-like growth factor (IGF)-I, IGF-II and IGF binding protein (IGFBP)-3 in schizophrenic Arab subjects. Clin Chem Lab Med. (2007) 45:1229–31. 10.1515/CCLM.2007.265 17635080

[17] Mabrouk H Houas I Mechria H Mechri A Douki W Gaha L. Oxidative stress markers in schizophrenic patients. Immun Anal Biol Spécial. (2013) 28:51–6. 10.1016/j.immbio.2012.10.00529710513

[18] Wen S Cheng M Wang HH Yue J Wang HH Li G Zheng L. Serum uric acid levels and the clinical characteristics of depression. Clin Biochem. (2012) 45:49–53. 10.1016/j.clinbiochem.2011.10.010 22040815

[19] Borovcanin M Jovanovic I Radosavljevic G Djukic Dejanovic S Bankovic D Arsenijevic N. Elevated serum level of type-2 cytokine and low IL-17 in first episode psychosis and schizophrenia in relapse. J Psychiatr Res. (2012) 46:1421–6. 10.1016/j.jpsychires.2012.08.016 22974591

[20] Borovcanin M Jovanovic I Radosavljevic G Djukic Dejanovic S Stefanovic V Arsenijevic N. Antipsychotics can modulate the cytokine profile in schizophrenia: attenuation of the type-2 inflammatory response. Schizophr Res. (2013) 147:103–9. 10.1016/j.schres.2013.03.027 23602340

[21] World Health Organization. International Statistical Classification of Diseases and Related Health Problems Tenth Revision. Geneva: World Health Organization (1992). 3376487

[22] Kay SR Fiszbein A Opler LA. The Positive and Negative Syndrome Scale (PANSS) for schizophrenia. Schizophr Bull. (1987) 13:261–76. 10.1093/schbul/13.2.261 3616518

[23] Sommer IEC Slotema CW Daskalakis ZJ Derks EM Dirk Blom J Van Der Gaag M. The treatment of hallucinations in schizophrenia spectrum disorders. Schizophr Bull. (2012) 38:704–14. 10.1093/schbul/sbs034 22368234 PMC3577047

[24] He Q You Y Yu L Yao L Lu H Zhou X. Uric acid levels in subjects with schizophrenia: a systematic review and meta-analysis. Psychiatry Res. (2020) 292:113305. 10.1016/j.psychres.2020.113305 32702552

[25] Yao JK Reddy R van Kammen DP. Reduced level of plasma antioxidant uric acid in schizophrenia. Psychiatry Res. (1998) 80:29–39. 10.1016/S0165-1781(98)00051-1 9727961

[26] Malewska-Kasprzak M Permoda-Osip A Rybakowski J. Disturbances of purinergic system in affective disorders and schizophrenia. Psychiatr Pol. (2019) 53:577–87. 10.12740/PP/97335 31522198

[27] Luo Q Wen Z Li Y Chen Z Long X Bai Y. Assessment causality in associations between serum uric acid and risk of schizophrenia: a two-sample bidirectional mendelian randomization study. Clin Epidemiol. (2020) 12:223–33. 10.2147/CLEP.S236885 32161502 PMC7049772

[28] Haj Mouhamed D Ezzaher A Neffati F Douki W Gaha L Najjar MF. Effect of cigarette smoking on plasma uric acid concentrations. Environ Health Prev Med. (2011) 16:307–12. 10.1007/s12199-010-0198-2 21431788 PMC3156839

[29] Liu F Du G-L Song N Ma Y-T Li X-M Gao X-M. Hyperuricemia and its association with adiposity and dyslipidemia in Northwest China: results from cardiovascular risk survey in Xinjiang (CRS 2008–2012). Lipids Health Dis. (2020) 19:58. 10.1186/s12944-020-01211-z 32238146 PMC7115071

[30] Choi HK Atkinson K Karlson EW Curhan G. Obesity, Weight Change, hypertension, diuretic use, and risk of gout in men. Arch Intern Med. (2005) 165:742. 10.1001/archinte.165.7.742 15824292

[31] Lanaspa MA Andres-Hernando A Kuwabara M. Uric acid and hypertension. Hypertens Res. (2020) 43:832–4. 10.1038/s41440-020-0481-6 32514150 PMC10000019

[32] Hu X Rong S Wang Q Sun T Bao W Chen L. Association between plasma uric acid and insulin resistance in type 2 diabetes: a Mendelian randomization analysis. Diabetes Res Clin Pract. (2021) 171:108542. 10.1016/j.diabres.2020.108542 33227361

[33] Lu Z Wen T Wang Y Kan W Xun G. Peripheral non-enzymatic antioxidants in patients with schizophrenia: a case-control study. BMC Psychiatry. (2020) 20:241. 10.1186/s12888-020-02635-8 32414343 PMC7227358

[34] Godin O Leboyer M Gaman A Aouizerate B Berna F Brunel L. Metabolic syndrome, abdominal obesity and hyperuricemia in schizophrenia: results from the FACE-SZ cohort. Schizophr Res. (2015) 168:388–94. 10.1016/j.schres.2015.07.047 26255568

[35] Pae C-U Paik I-H Lee C Lee S-J Kim J-J Lee C-U. Decreased plasma antioxidants in schizophrenia. Neuropsychobiology. (2004) 50:54–6. 10.1159/000077942 15179021

[36] Xuan J Pan G Qiu Y Yang L Su M Liu Y. Metabolomic profiling to identify potential serum biomarkers for schizophrenia and risperidone action. J Proteome Res. (2011) 10:5433–43. 10.1021/pr2006796 22007635

[37] Palmgren K Wighton A Reynolds CW Butler A Tweed JA Raniwalla J. The safety and efficacy of zotepine in the treatment of schizophrenia: results of a one-year naturalistic clinical trial. Int J Psychiatry Clin Pract. (2000) 4:299–306. 10.1080/13651500050517867 24926581

[38] Kolls JK Lindén A. Interleukin-17 family members and inflammation. Immunity. (2004) 21:467–76. 10.1016/j.immuni.2004.08.018 15485625

[39] Storelli E Cassina N Rasini E Marino F Cosentino M. Do Th17 Lymphocytes and IL-17 contribute to Parkinson's disease? A systematic review of available evidence. Front Neurol. (2019) 10:13. 10.3389/fneur.2019.00013 30733703 PMC6353825

[40] Moser T Akgün K Proschmann U Sellner J Ziemssen T. The role of TH17 cells in multiple sclerosis: therapeutic implications. Autoimmun Rev. (2020) 19:102647. 10.1016/j.autrev.2020.102647 32801039

[41] Huppert J Closhen D Croxford A White R Kulig P Pietrowski E. Cellular mechanisms of IL-17-induced blood-brain barrier disruption. FASEB J. (2010) 24:1023–34. 10.1096/fj.09-141978 19940258

[42] Huang Y-YY Ye Z Gu S-WW Jiang Z-YY Zhao L. The efficacy and tolerability of febuxostat treatment in a cohort of Chinese Han population with history of gout. J Int Med Res. (2020) 48:030006052090295. 10.1177/0300060520902950 32363973 PMC7221481

[43] Yao JK Condray R Dougherty GG Keshavan MS Montrose DM Matson WR. Associations between purine metabolites and clinical symptoms in schizophrenia. PLoS ONE. (2012) 7:e42165. 10.1371/journal.pone.0042165 22916123 PMC3419238

[44] Tao Q Miao Y Li H Yuan X Huang X Wang Y. Insulin resistance and oxidative stress: In relation to cognitive function and psychopathology in drug-naïve, first-episode drug-free schizophrenia. Front Psychiatry. (2020) 11:537280. 10.3389/fpsyt.2020.537280 33329081 PMC7732418

[45] Coleman M. A crossover study of allopurinol administration to a schizophrenic child. J Autism Child Schizophr. (1974) 4:231–40. 10.1007/BF02115229 4613729

[46] Green HF Khosousi S Svenningsson P. Plasma IL-6 and IL-17A correlate with severity of motor and non-motor symptoms in Parkinson's disease. J Parkinsons Dis. (2019) 9:705–9. 10.3233/JPD-191699 31524180 PMC6839458

[47] Veselý B Koritáková E Bohnen NI Viszlayová D Királová S Valkovič P. The contribution of cerebrovascular risk factors, metabolic and inflammatory changes to cognitive decline in Parkinson's disease: preliminary observations. J Neural Transm. (2019) 126:1303–12. 10.1007/s00702-019-02043-7 31332506 PMC6959128

[48] Solberg DK Refsum H Andreassen OA Bentsen H. A five-year follow-up study of antioxidants, oxidative stress and polyunsaturated fatty acids in schizophrenia. Acta Neuropsychiatr. (2019) 31:202–12. 10.1017/neu.2019.14 31178002

